# Hybrid Genetic Algorithm for Clustering IC Topographies of EEGs

**DOI:** 10.1007/s10548-023-00947-y

**Published:** 2023-03-07

**Authors:** Jorge Munilla, Haedar E. S. Al-Safi, Andrés Ortiz, Juan L. Luque

**Affiliations:** 1 Dpto. Ingeniería de Comunicaciones, Universidad de Málaga, Campus de Teatinos, 29071 Málaga, Málaga Spain; 2 Dpto. Psicología Evolutiva y Educación, Universidad de Málaga, Campus de Teatinos, 29071 Málaga, Málaga Spain

**Keywords:** Clustering, EEG, ICA, GA

## Abstract

Clustering of independent component (IC) topographies of Electroencephalograms (EEG) is an effective way to find brain-generated IC processes associated with a population of interest, particularly for those cases where event-related potential features are not available. This paper proposes a novel algorithm for the clustering of these IC topographies and compares its results with the most currently used clustering algorithms. In this study, 32-electrode EEG signals were recorded at a sampling rate of 500 Hz for 48 participants. EEG signals were pre-processed and IC topographies computed using the AMICA algorithm. The algorithm implements a hybrid approach where genetic algorithms are used to compute more accurate versions of the centroids and the final clusters after a pre-clustering phase based on spectral clustering. The algorithm automatically selects the optimum number of clusters by using a fitness function that involves local-density along with compactness and separation criteria. Specific internal validation metrics adapted to the use of the absolute correlation coefficient as the similarity measure are defined for the benchmarking process. Assessed results across different ICA decompositions and groups of subjects show that the proposed clustering algorithm significantly outperforms the (baseline) clustering algorithms provided by the software EEGLAB, including CORRMAP.

## Introduction

EEG recordings are used in clinical and cognitive brain research. Comparative across subjects using directly such scalp-recorded EEG signals poses some problems because they are a mixture of an unknown number of brain and no-brain contributions, and therefore the spatial relationship of the physical electrode site to the underlying cortical areas that summed generate such activity may be rather different in different subjects, depending on the physical locations, extents, and particularly the orientation of the cortical source areas, both in relation to the own active electrode site and its reference channel. A way to circumvent this issue is the use of independent component analysis (ICA) (Bell and Sejnowski 1995). ICA is nowadays an essential method for the processing of EEG signals, particularly for the removal of artifacts. ICA is a blind source separation algorithm that performs a linear un-mixing of multi-channel EEG recording into maximally temporally independent statistical source signals, which are further referred to as independent components (ICs), and which represent brain and non-brain (artifact) processes.

There is not a straightforward way to identify equivalent components across subjects so that an effective way to assess the reliability of the results of an EEG-based experiment is studying IC clusters. A typical goal is to find clusters of brain-generated IC processes associated more frequently with the population of interest. When external information about the labels is available, supervised or semi-supervised methods (Yin et al. 2012; Piroonsup and Sinthupinyo 2018) can be applied, but in most real-world cases, these external information is not present and clustering of ICs is a challenging unsupervised learning task that requires well-defined internal validation metrics.

ASSR (Auditory steady-state response) EEGs measure the response that is evoked by a periodically repeated auditory stimulus (Farahani et al. 2021; Hwang et al. 2020). This kind of neurophysiological response has been used successfully to study patients with schizofrenia (Koshiyama et al. 2021), bipolar disorder, depression and autism (Jefsen et al. 2022) and, more recently, developmental dyslexia (Gallego-Molina et al. 2022). Event-Related Potentials (ERPs) are not available in those cases so that clustering of ICs must be tackled using other features such as spectra-time frequency results or source-localization (e.g. (Lin et al. 2011)). However, some authors, such as (Miyakoshi 2023), advice against the use of multiple clustering criteria and recommend the use of source locations, and others (e.g. (Viola et al. 2009)) point out that the joint use of features leaves the user with a choice of weights which is not easy to address. Thus, (Artoni et al. 2014, 2018) and CORRMAP (Viola et al. 2009) propose the use of correlation coefficient between IC time courses and IC topographies, respectively. IC topographies, or also termed as (2D) scalp or topographic maps, coincide, as we will show later, with the inverse weight returned by the ICA analysis. More recently, (de Meneses et al. 2022) has proved the effectiveness of the use of topographic maps for supervised group classification using Convolutional Networks. CORRMAP, which is the most popular clustering method for IC scalp maps, works as an open-source plugin for the popular EEGLAB software (Delorme and Makeig 2004), which further provides the possibility of clustering IC scalp maps using other clustering algorithms. To the best of our knowledge, these are the most currently used clustering algorithms for topographic maps, so their results will be used as the baseline performances for benchmarking.

This paper presents a new hybrid genetic algorithm for the clustering of IC topographies along with the definition of internal validation metrics to assess and compare their results. The new clustering algorithm implements two genetic algorithms (GA): one for the computation of the polarity inversion of the components before computing the average image of the clusters (centroids) and another for getting the final partitional clustering. The algorithm automatically estimates the number of clusters using a fitness function that incorporates local-density aspects. This algorithm is defined as hybrid since the initialization values of the second GA are provided by a pre-clustering phase. This pre-clustering phase is based on spectral-clustering, allowing a direct adaptation from the pairwise absolute correlation coefficients. The proposed algorithm outperforms the results provided by the most currently used clustering methods when these are assessed across ICA decompositions and groups of subjects.

The rest of this paper is organized as follows. Section 2 describes the database used and the methods followed for the development of this work, including the metrics applied for benchmarking. Section 3 reviews the clustering methods for IC topographies and Sect. 4 introduces the proposed algorithm. Section 5 assesses these results across different ICA decompositions and groups of subjects. Lastly, Sect. 6 discusses the main conclusions.

## Materials and Methods

This work uses the EEG data obtained by the Leeduca research group at the University of Málaga (Ortiz et al. 2020). Forty-eight participants took part in the study by the Leeduca Study Group. These subjects were matched in age (t(1) = $-$1.4, p > 0.05, age range 88–10 months). The participants were 32 skilled readers (17 males) and 16 dyslexic readers (7 males). The control group had a mean age of 94.1 ± 3.3 months, and the dyslexic group 95.6 ± 2.9 months. The experiment was conducted in the presence of each child’s legal guardians and with their understanding and written consent.

EEG signals were recorded using the Brainvision actiCHamp Plus with actiCAP (Brain products GmbH, Germany). It had 32 active electrodes and was set at a sampling rate of 500Hz. These electrodes were located in the 10–20 standardized system. Participants underwent 15-minute sessions in which they were presented white noise auditory stimuli modulated at 4.8, 16, and 40Hz sequentially in ascending and descending order, for 2.5 min each. Participants were right-handed, native Spanish speakers. They had a normal or corrected-to-normal vision and no hearing impairments.

For the analysis carried out in this paper, just a sample of the complete study has been taken; more specifically, we have selected the EEGs corresponding to subjects of the control group under the stimuli of ascending 40Hz. The clustering have been carried out for sets of 5 subjects, which means 155 ICs each, looking for a compromise between enough complexity to test the clustering methods and convenience to represent and interpret the results.

**Fig. 1:**
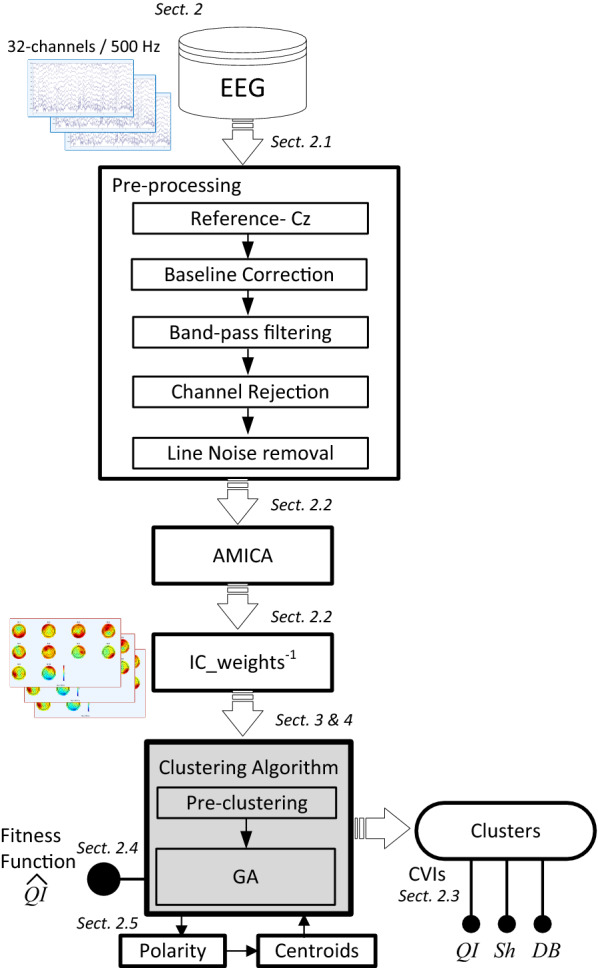
Workflow applied to EEG data. It also indicates the sections where the different steps are addressed throughout the paper

Figure 1 shows the workflow of the benchmark, indicating the processes applied to the recorded EEG signals to obtain the ICs, their clustering and the metrics to compare the results. The figure also includes the sections where each aspect is addressed throughout the paper.

### Signal Pre-processing

EEG signal pre-processing constitutes an important stage due to the presence of artifacts and the low signal-to-noise ratio of EEG signals. A prior pre-processing during recording of EEG signal consisted of removal of the most evident artifacts and the normalization of the duration to be 136-second segments (instead of 150 s). Then, the pre-processing of these segments, carried out using EEGLABv2021b, included the following steps:

1. Import EEG Data and channel location into EEGLAB. A *.sph* file with the Matlab spherical coordinates was initially created according to the 10–20 EEG Placement method used for the EEG recording. EEG Data, stored in a *.mat* file, were then imported along with the *.sph* file.
2. Signal from each channel was referenced to the Cz electrode. As a result, EEG data goes from having 32 to 31 channels.
3. Baseline correction was applied to every channel to remove possible temporal drifts and prevent artifacts when filtering in the next step. As dataset is continuous, channel means are removed separately.
4. Data were filtered using a high-pass filter (FIR type with cancellation of phase shift) with cut-off frequency of 1Hz, which is a recommended value to obtain good quality ICA decompositions (Klug and Gramann 2021). Although the selection of 1 Hz as the lower edge filters out part of the Delta band, this value is chosen to cope with the sensitivity of ICA algorithms to low-frequency shift and because event related potentials were not going to be processed. A low-pass filter (of the same type) with cut-off frequency of 50 Hz was applied then to keep the core part of the Gamma waves but reducing the overlap with the electromyographic frequency band (Muthukumaraswamy 2013).
5. Automatic Channel Rejection was applied using Kurtosis. The Kurtosis value is computed for each channel and outliers are determined using a z-score threshold of 10. This value is relatively high so that only seriously contaminated channels were rejected.
6. Line Noise removal was applied using an approach advocated in (Mitra and Bokil 2007) and implemented with the plug-in Cleanline.

### ICA Algorithm and IC Topographies

ICA is the most extended data-driven method for parsing EEG signals, combining brain and non-brain sources in the scalp electrodes, into a set of maximally temporally and functionally independent components (Bell and Sejnowski 1995). More formally, there are some source activities $\varvec{b}$ and we just know their projections on each electrode *x*, which record the mix of these activities because every neuronal source projects to most (or even all) electrodes. This effect is modeled by a mixing or transformation matrix *W*, where each column is referred to as the extraction filter or “weights”, so that $x=W \cdot b$. Thus, ICA (as a source blind separation) can be used to find the unmixing matrix *A*, with $A=W^{-1}$ provided that *W* is invertible. Otherwise, in the general case, the pseudoinverse is computed: $A=(W*SM)^{+}$, with *SM* the spherical matrix that reprojects the ICA solution back into the original coordinate frame to undo the whitening (or sphering) of the data, generally applied in the first step of the ICA algorithms as a way to force the different channels to be uncorrelated. The columns of *A* are referred to as activation patterns, or “inverse weights”, and encode the strength with which the source’s activity is present in each channel (Haufe et al. 2014), so they are used to represent the IC topographies or scalp maps.

In practice, computation of $\varvec{A}$, when noise and the rest of interferences are considered, is not trivial and different algorithms to compute ICA have been proposed which seek to maximize the statistical independence of the estimated components. The differences in the variable chosen to define the independence make these algorithms return somewhat different results when applied to the same EEG data. Infomax (Lee et al. 2000) is likely the most applied algorithm for EEG data but for this work we have used AMICA, which (slightly) outperforms Infomax in component separation (Delorme et al. 2012). 68,000 points are used for the computation, which is larger than the 30,800 ($\sim 32^2*30$) data points usually recommended for 32 channels (Miyakoshi 2023). The maximum number of learning steps is set to 1000.

The output of this step is a matrix of dimensions $31 \times 155$, corresponding to the 31 ICA inverse weights for the 31 channels (32 minus the reference) of the EEG recordings of 5 subjects.

### Quality Metrics

**Fig. 2:**
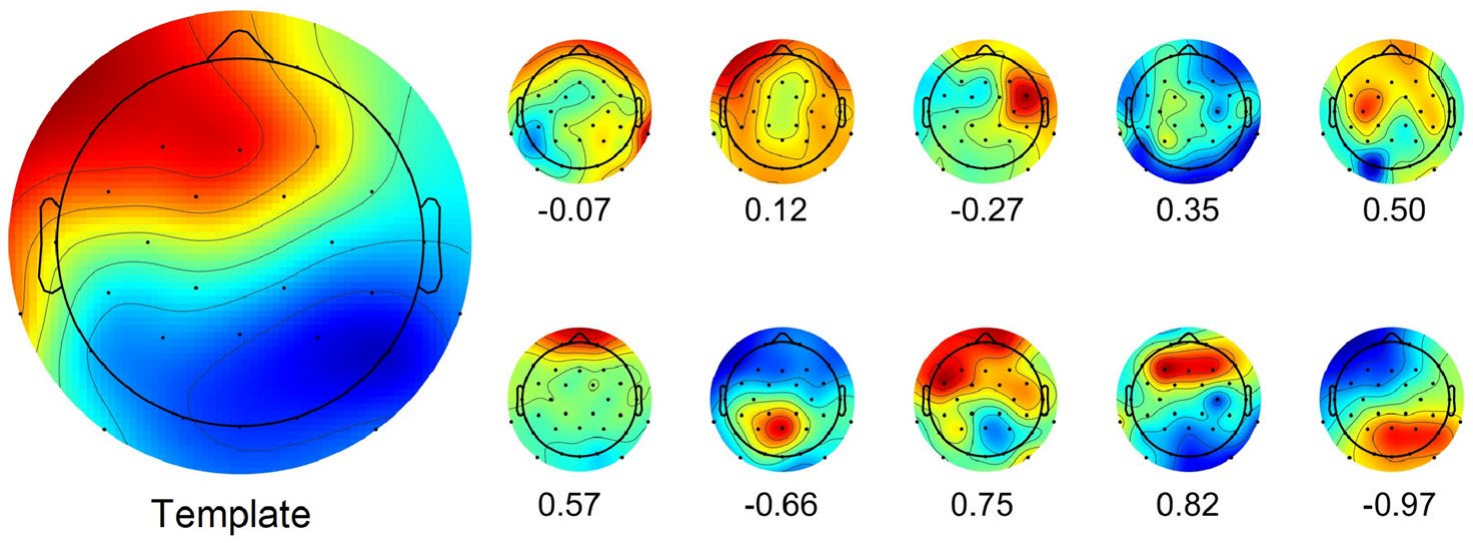
Scalp maps of ICs with different correlation coefficients with an IC template

Component scalp maps have no absolute polarity, which is known as the sign ambiguity problem (Onton et al. 2006). Thus, the absolute correlation coefficients between the inverse weights computed by AMICA are used as the similarity measure (Viola et al. 2009). Figure 2 shows an IC template and different ICs with different values of correlation coefficient (negative values correspond to inverted polarity).

The goal of the clustering algorithms is dividing ICs into clusters such that scalp maps of ICs within the same cluster are similar while those in different clusters are distinct. External information is not available here (unsupervised learning task), so it is necessary to find a way to validate the goodness of these partitions. In the literature, a number of internal clustering validation measures have been proposed (Liu et al. 2012), presenting all of them certain limitations in the different application scenarios (e.g. presence of noise, density differences, arbitrary cluster shapes (Sheng et al. 2005)). To the best of our knowledge, the closest clustering validity index (CVI) to this scenario is the Quality Index used in (Artoni et al. 2018, 2014), which is further inspired by the Calinski-Harabasz criterion (Caliński and Harabasz 1974), defined as the difference between the average within-cluster similarities and the average between-cluster similarities:

1 $$\begin{aligned} QIc_m = 100*\biggr [\frac{1}{ |{C_m}|^2-|{C_m}|} \sum \limits _{\begin{array}{c} i,j \in {\text { }C_m}\\ i \ne j \end{array}} |{\varvec{R}_{ij}}|- \frac{1}{|{C_m}||{C_{- m}}|} \sum \limits _{i \in {\text { }}{C_m}} \sum \limits _{j \in {\text { }}{C_{-m}}} |{\varvec{R}_{ij}}|\biggr ] \end{aligned}$$

where $C_m$ denotes the set of ICs that belong to the *m*-th cluster, and $C_{-m}$ the set of ICs that do not, $|\varvec{R}_{ij}|$ the similarity between the *i*-th and *j*-th ICs (i.e. the absolute correlation coefficient between inverse ICA weights in this case), and $|\mathcal {S} |$ the cardinality of the set $\mathcal {S}$. The more compact the cluster, the higher the *QIc*. Then, the overall quality of a clusterization, with *k* clusters, is computed as the weighted average of the *QIc*, with weights proportional to the size of each cluster:

2 $$\begin{aligned} QI =\sum \limits _{m = 1}^k {\frac{{ {|{{C_m}} |} }}{{{|{{C_m}} |} + |{C_{-m}}|}} QIc_{m}}. \end{aligned}$$

This index is complemented here with the adaptation to this specific similarity measure of two of the most important CVIs: the silhouette graph (Rahim et al. 2021) and the Davies-Bouldin index (Davies and Bouldin 1979).

Silhouette graphs represent the indexes $s_i$ for each component, computed as follows:

3 $$\begin{aligned} s_i=(a_i-b_i)/max(a_i,b_i), \end{aligned}$$

with $a_i$ the average of the absolute correlation coefficients from the *i*-th IC (in cluster $C_m$) to the other ICs in the same cluster:

4 $$\begin{aligned} a_i=\frac{1}{ |{C_m}|} \sum \limits _{\begin{array}{c} j \in {\text { }C_m}\\ i \ne j \end{array}} |{\varvec{R}_{ij}}|, \end{aligned}$$

and $b_i$ the maximum average absolute correlation coefficient value of the *i*-th IC to ICs in a different cluster, maximized over clusters:

5 $$\begin{aligned} b_i =\begin{array}{c} max\\ n \ne m \end{array} \left( \frac{1}{ |{C_n}|} \sum \limits _{j \in {\text { }C_n}} |{\varvec{R}_{ij}}|\right). \end{aligned}$$

The function $max(a_i,b_i)$ returns the maximum value between $a_i$ and $b_i$, so that the silhouette values range from -1 to 1. Finally, the Silhouette CVI, denoted by *Sh*, is computed as the average of the $s_i$ excluding noisy ICs. Noisy ICs, or outliers, are those ICs that are not assigned to any cluster, being included within the cluster “others”.

Davies-Bouldin index provides a rate between compactness and separation. Compactness for the *m*-th cluster, with centroid *M*, is computed as:

6 $$\begin{aligned} d_m =\frac{1}{|{C_m}|}\sum \limits _{i \in {\text { }C_m} } |R_{iM} |, \end{aligned}$$

and the separation as the similarity between the centroids of the different clusters: $\vert R_{MN} \vert$, which stands for the similarity between the centroid of the clusters *m* and *n*. Then, the Davies-Bouldin CVI is computed, for a total of *k* clusters, as follows:

7 $$\begin{aligned} DB =\frac{1}{k}\sum \limits _{m=1}^{k} DB_m, \end{aligned}$$

with:

8 $$\begin{aligned} DB_m =\begin{array}{c} max\\ m \ne n \end{array} \left(\frac{|R_{MN} |}{d_m + d_n}\right), \end{aligned}$$

where it must be noted that this rate has been inverted regarding the traditional definition of the index to adapt it to the similarity measure used here. This way, likewise the original index, $DB_m$ represents the worst-case within-to-between cluster ratio for cluster *m* and the optimal clustering solution has the smallest *DB* index value.

Finally, although the validation of the clusterings is internal, a CVI inspired by the rand index (*RI*) (Gates and Ahn 2017) is used to compare the results of the different clustering with those provided by the ICLabel algorithm (Pion-Tonachini et al. 2019a), described in the next section. This modified rand index, *RIm*, is properly defined later.

### Fitness Function

The fitness function used in the GA is based on the CVIs introduced in the previous section but modified with local information (Tinós et al. 2018). More specifically, this cost function $\widehat{QI}$ is based on the *QI* defined in the previous section but the different subfunctions $\widehat{QIc}_{m}$ do not depend on all the objects but just on a reduced number of objects, so that neighborhood relations are used to compute the functions. Thus, for computing the between-cluster similarities, each function $\widehat{QIc}_{m}$ is influenced by the objects in the *m*-th cluster and by a subset of the $|C_m |Fc$ closest objects; i.e. the highest similarities in terms of absolute correlation coefficient, where $F_c$ is a regularization factor. More formally,

9 $$\begin{aligned} \begin{aligned} \widehat{QIc}_m = 100*\biggr [ \frac{1}{\vert C_m \vert ^2-\vert C_m \vert }\sum \limits _{\begin{array}{c} i,j \in {\text { }C_m}\\ i \ne j \end{array}} {\vert \varvec{R}_{ij}} \vert \ldots \\ - \frac{1}{\vert C_m \vert F_c}\sum \limits _{p=1}^{\vert C_m \vert F_C} {{sort_p(\vert \varvec{R}_{ij} \vert , \, \forall i \in {C_m} \text { and } \forall j \in {C_{ - m}}} } \biggr ] \end{aligned} \end{aligned}$$

where $sort_p$ sorts in descending order the between-cluster similarities. For the experiments in this paper, *Fc* has been set to 10).

### Computation of Centroids

The computation of the centroid, or average scalp map, of each cluster requires, as a consequence of the sign ambiguity, to determine firstly the polarity inversions to apply to the ICs. These polarity inversions can be represented as a vector $\varvec{s}\in \{-1,1\}^{n}$, with *n* the number of ICs. CORRMAP and EEGLAB use references to determine the polarities of the different ICs as the sign of the correlation coefficients of these with such reference. This method, however, presents problems when the reference has low absolute correlation values with some of the elements. A simple toy example which illustrates this point is presented next. Let us assume that IC1 is selected as template (reference) for the following correlation coefficient matrix between the ICs:

$$\begin{aligned} \varvec{R} = \left[ {\begin{array}{*{20}{c}} 1&\quad{}{0.1}&\quad{}{ - 0.1}&\quad{}{-0.8} \\ {0.1}&\quad{}1&\quad{}{0.9}&\quad{}{-0.5} \\ { - 0.1}&\quad{}{0.9}&\quad{}1&\quad{}{ -0.3} \\ {-0.8}&\quad{}{-0.5}&\quad{}{ -0.3}&\quad {}1 \end{array}} \right] . \end{aligned}$$

Thus, the vector $\varvec{s}\in \{-1,1\}^4$ that determines the polarity inversion would be $\varvec{s}^1=[1\; 1\; \text{-1 } \; \text{-1 }]$, implying that IC2 and IC3 would be subtracted, which does not seem to be correct since the correlation between them is high and positive. In the proposed algorithm, this problem is addressed by analyzing the polarity inversions globally with the implementation of a GA that seeks to find a polarity inversion vector $\varvec{s}$ which minimizes the following cost function:

10 $$\begin{aligned} Cost=0.5 |\varvec{s^T}*\varvec{s}-\varvec{S} |.* \Vert \varvec{R} \Vert _1 \end{aligned}$$

where.* denotes element-by-element multiplication, $||\varvec{R}||$ the entrywise L1-norm on matrix $\varvec{R}$ and $\varvec{S}$ the matrix with the signs of $\varvec{R}$; i.e. $\varvec{S}_{i,j}=1$ if $\varvec{R}_{i,j}$ is positive, and -1 otherwise. This cost function has a value 2.4 for the previous $\varvec{s}^1$, while it is just 0.2 for the optimal solution computed with the algorithm: $\varvec{s}^2=[ 1 \; 1 \; 1 \; \text{-1 }]$ (and its complementary [-1  -1  -1  1]). For more details about the implementation of this GA, we refer the reader to (Munilla et al. 2022).

## Clustering Algorithms for IC Topographies

This section reviews the main clustering methods for scalp maps, showing the results for the 155 previously computed ICs. Next section assesses the results across different ICA decompositions and groups of subjects. It is also worth to mention that other clustering algorithms not specifically intended for this purpose, such as DBSCAN (Schubert et al. 2017), have been also tried, with conversion from correlations to distances when required, but the results obtained were not relevant enough to be included here.

### ICLabel

**Fig. 3:**
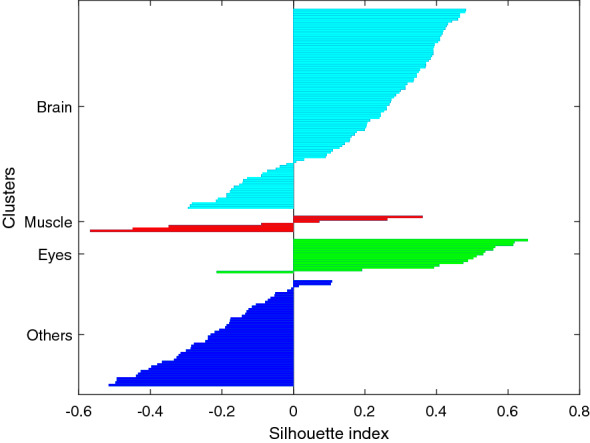
Silhouette graph for ICLabel

ICLabel classifier is an EEG IC classifier that has shown to perform very well estimating IC classifications as compositional vectors across seven IC categories (Pion-Tonachini et al. 2019b): 1) brain, activity believed to originate from locally synchronous activity in one (or two well-connected) cortical patches; 2) muscle, high-frequency and broadband components, above 20–30Hz, originated from groups of muscle motor units; 3) eye, which activity originating from the eyes and which can be further subdivided into ICs accounting for activity associated with horizontal eye movements and ICs accounting for blinks and vertical eye movements; 4) heart, they are quite rare and are related to the fact of placing an electrode directly above a superficial vein or artery; 5) Line Noise, concentrated at 50/60Hz and which captures the effects of line current noise emanating from nearby electrical fixtures o poorly grounded EEG amplifiers; 6.) Channel Noise, indicating that some portion of the signal recorded at an electrode channel is already nearly statistically independent of those from other channels; and 7) Others, which catches those ICs that fit none of the previous types. Thus, ICLabel provides us with a rough initial clustering along with an “estimated” label for the IC components. For this particular case, it results in 3 clusters with: 87 brains, 7 muscles and 15 eyes, leaving 46 as others. The silhouette graph is plotted in Fig. 3, corresponding to $Sh=0.21$, and $QI_c$ of 10.2, 1 and 23 for the clusters Brain, Muscle and Eye, respectively ($-$1.7 for others), with *QI*=8 and $\widehat{QI}$=7.6.

### CORRMAP

CORRMAP finds scalp maps that are similar to another selected by the user as a template. It is thus defined as a semi-automatic clustering tool because it does not directly provide the clusters but finds IC topographies that are similar to an user-defined template. The core of the algorithm is a two-step loop. In the first step, the absolute correlation coefficients between a selected IC (template) and the rest of ICs from all datasets are computed. For each dataset, CORRMAP selects up to a number *g*, chosen by the user from 1 to 3, of ICs with the largest supra threshold absolute correlation with the template. Next, an average cluster map is calculated, after inversion of those ICs showing a negative correlation with the template and root mean square (RMS) normalization of each IC. In the second step, the process is repeated but using the average cluster map obtained in the first step as the new template.

To evaluate CORRMAP clustering performances, we run it 155 times by selecting every IC as the template and looking for similar ICs across subjects (with automatic threshold). A symmetric adjacency matrix is then built setting an edge between the IC selected as template and the ICs found by CORRMAP for such template.

**Fig. 4:**
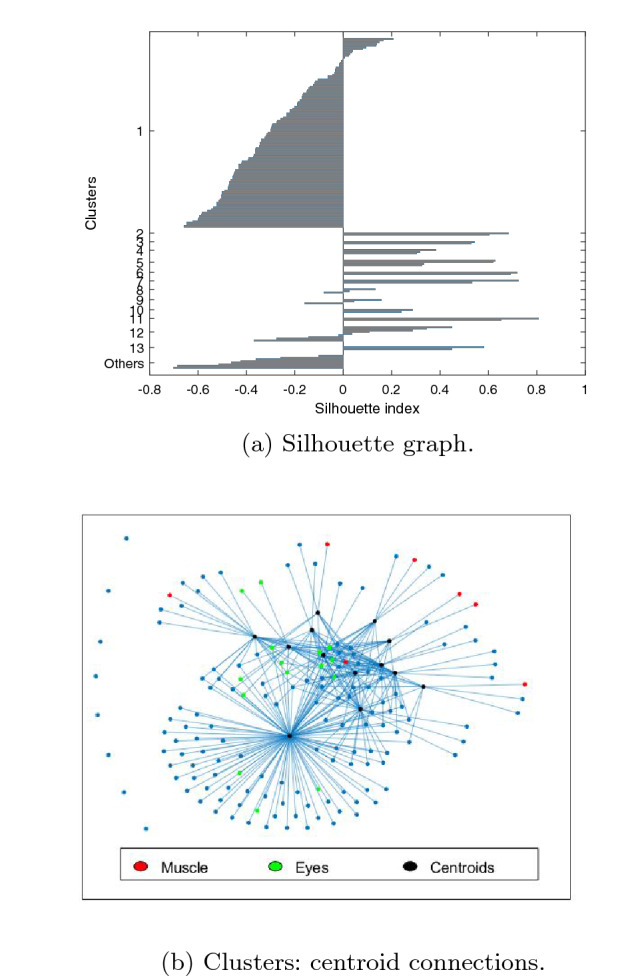
Results using CORRMAP

The best results are obtained for $g=1$. Figure 4 shows the silhouette graph of the obtained clustering, with *Sh*=$-$0.1, and a graph with the centroid connections. In the latter, clusterization are represented as a graph, with an edge between the centroid *m*, computed as the average image of the cluster $C_m$, and any node *i* provided that $\vert R_{mi} \vert \ge min( \vert R_{mj} \vert )$ for $\forall j \in C_m$, revealing the relationship between clusters. The figure allows checking at a glance the number of clusters, number of outliers, size of the clusters and separation of the different clusters. Ideally, each element should be connected to a single centroid and each centroid exclusively to the elements of its cluster. Additionally, the figure points out with different colors those ICs labelled by IClabel as eyes (green) or muscle (red), so it is possible to check how these have been clustered. A total of 13 clusters are obtained with *QI*=15.8, $\widehat{QI}$=11.3 and 8 outliers. It must be noted that there is a big cluster of 111 ICs, which makes even bigger when *g* increases (125 for $g=2$ and 127 for *g*=3).

### PCA-Based Built-In EEGLAB Clustering Algorithms

EEGLAB implements some PCA-based clustering algorithms that allow clustering EEG data according to different characteristics, including the scalp map similarities. The performances provided K-means (Statistics Toolbox), Neural Network and K-meansCluster (Non-Statistic toolbox), henceforth K-meansC, are analyzed here. These methods do not work directly on the inverse ICA weights but on their corresponding topographic map (67x67 matrices).

**Fig. 5:**
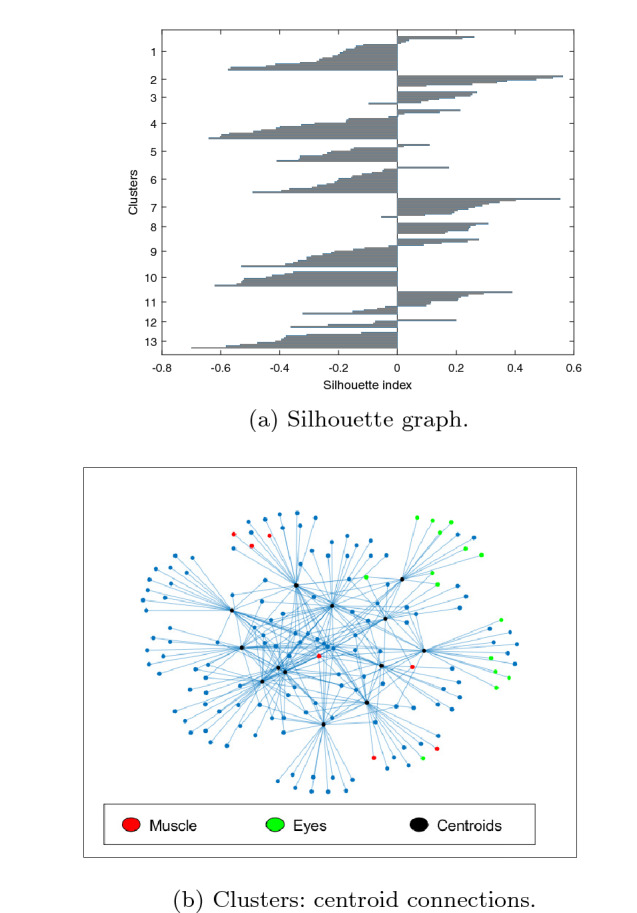
Results using Kmeans of EEGLAB

A grid search for the optimal number of clusters *k* and number of PCA components *p* is carried out between 10 and 18, and 3 and 11, respectively, and averaging on 10 realizations for K-means and Neural Network as these algorithms use random initial seeds. The best results, in terms of the cost function $\widehat{QI}$, and the parameters used in each case are described next. For K-means, $QI=14\pm 0.6$ and $\widehat{QI}=9.8\pm 0.5$ are obtained for $k=13$, $p=7$ and separating as outliers those components to more than 3 standard deviations. For Neural Networks, the best results are obtained for $k=10$ and $p=11$: $QI=10.7\pm 0.8$ and $\widehat{QI}=9\pm 1.2$. And finally, for K-meansC, $QI=14$ and $\widehat{QI}=$9.3 are obtained for $k=14$ and $p=6$. Figure 5 shows the silhouette and the centroid connections for K-means (one of the realizations).

## Novel Clustering Algorithm

**Fig. 6:**
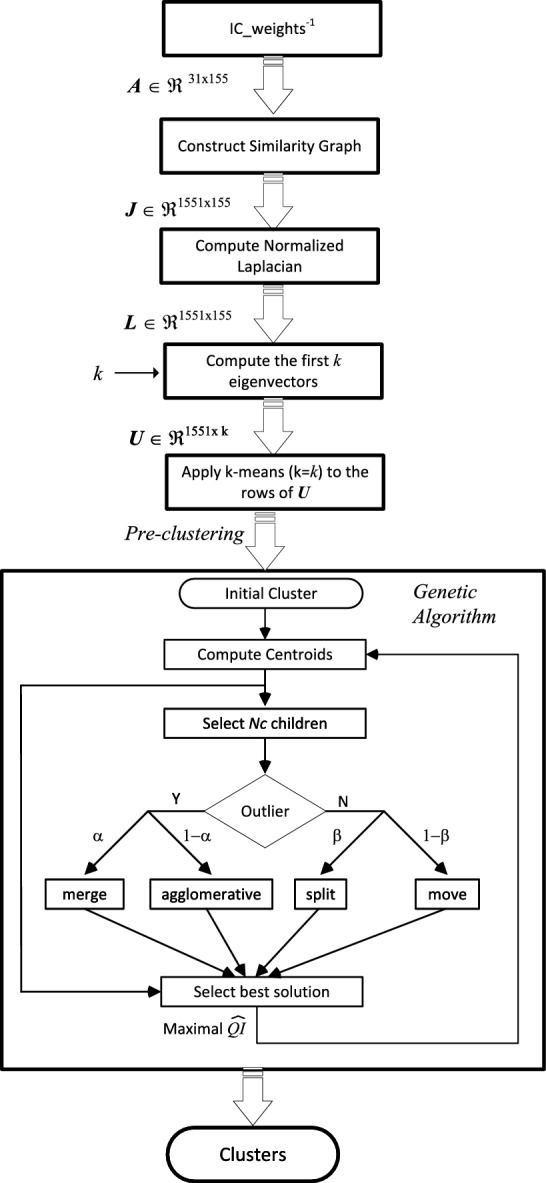
Flowchart of the proposed clustering algorithm

Figure 6 shows the flowchart of the novel clustering algorithm proposed in this paper. A pre-clusterization based on spectral clustering is followed by a clustering genetic algorithm (CGA). Spectral clustering (Ng et al. 2001) is not based on distance but in similarity graphs, which makes it particularly suitable for this case where the absolute correlation coefficient is used as similarity measure. Thus, the proposed clustering algorithm starts by computing a similarity graph *G* as an undirected graph where the edges between two vertices (ICs) carry a non-negative weight proportional to the similarity measure. More exactly, the adjacency matrix $\textbf{J}$ of *G* is computed as: $\textbf{J}=\vert \textbf{R} \vert$ (see Sect. 2.3). Then the symmetric normalized Laplacian is computed (Chung 1997):

11 $$\begin{aligned} \textbf{L}=\textbf{I}-\textbf{D}^{-1/2}\textbf{J}\textbf{D}^{-1/2} \end{aligned}$$

where $\textbf{D}$ is the degree matrix of *G*.

The next step is computing the first *k* eigenvectors of $\textbf{L}$, corresponding to the *k* smallest eigenvalues. The value *k* is an input parameter of the algorithm and will determine the initial number of pre-clusters. For a first approach to this value, the eigenvalues can be used. From the Laplacian properties, the number of eigenvalues which are (approximately) zero coincides with the number of components (independent clusters) in the graph (von Luxburg 2007). The first nonzero eigenvalue is called the eigengap or spectral gap, and informs us about the connectivity of the graph and the number of clusters. As explained later, the final number of clusters may change after applying the second optimization phase. The *k* smallest eigenvectors are then arranged in columns to have a matrix $\textbf{U} \in \mathbb {R}^{n \times k}$, with *n*=155 in this case. Using this, the *n* vectors $y_i \in \mathbb {R}^k$ corresponding to the rows of $\textbf{U}$ are clusterized in *k* clusters to get the pre-clustering. These vectors $y_i$ are the coordinates of the original data points in a lower-dimensional space created by the selected eigenvectors. This change of the representation of the data points from $\mathbb {R}^{n}$ to $\mathbb {R}^{k}$ makes clustering easier and is the “key” of the spectral-clustering. Thus, for this step, a simple K-means without outliers algorithm has been employed. Finally, each original point *i* is assigned to the same cluster that its representation $y_i$ in the reduced dimensional space.

The clustering obtained in the previous phase is then used as the initial seed for the GA implemented in the next phase. This is an elitist algorithm that implements centroid-based encoding. Integer encoding with a vector of *N*=155 positions is employed (Hruschka et al. 2009), allowing that the number of clusters can change during the optimization process. A population of *Nc* children is generated by randomly selecting one of the *N* objects (ICs). For each of these children, one of four possible mutation operator is employed: *merge* and *agglomerative* if the selected IC is an outlier, and *split* and *move*, otherwise. The operators *merge* and *agglomerative*, applied with probability $\alpha$ and $1-\alpha$, respectively, join the selected IC, which currently is an outlier, to the closest outlier (provided that it exists) to form a new cluster (*merge*) or to the cluster with the closest centroids (*agglomerative*). The operators *split* and *move*, applied with probability $\beta$ and $1-\beta$, respectively, assign the selected IC to the group of outliers (*split*) or the cluster with the closest centroid (*move*). The *Nc* resulting clusterings are evaluated and compared with the initial seed. The best solution is chosen as seed for the next iteration. The process is repeated for up to a maximum of iterations, *MaxIter*, or the results are not improved for a certain number of iterations $\Delta Iter$. This optimization automatically returns the optimum number of clusters provided that *k* in the pre-clustering phase is chosen within a certain range.

**Fig. 7:**
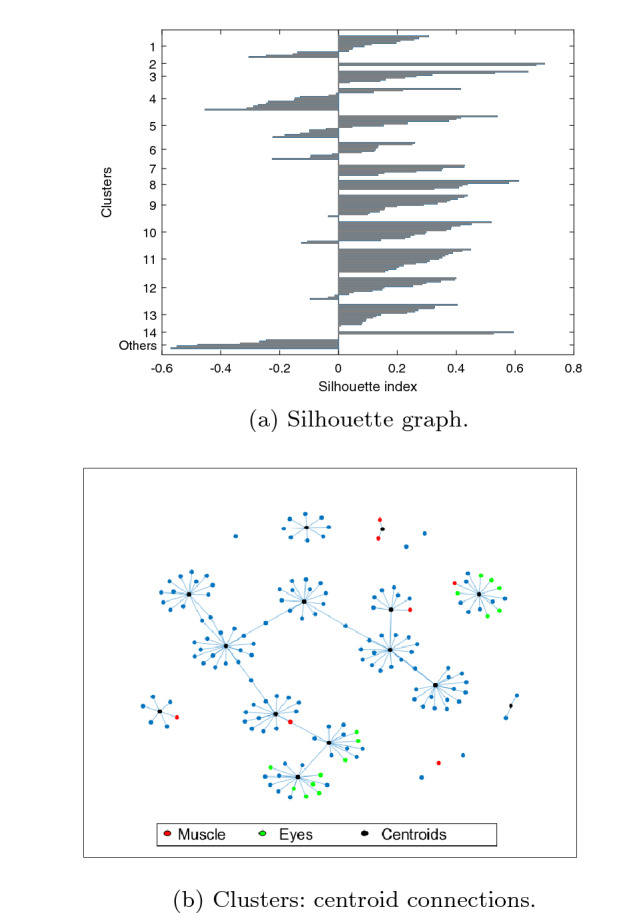
Results using the proposed method

For these input parameters: $N=155$, $k=13$, $Nc=20$, $\alpha =0.5$, $\beta =0.5$, $MaxIter=5000$ and $\Delta Iter$=250, Fig. 7 shows the silhouette graph ($SH=0.18\pm 0.01$) and the centroid connections. The number of final clusters is $13.9 \pm 0.8$ clusters (values from *k* between 9 and 15 converge to around 14), the ICs classified as others is $4.7 \pm 1.5$, $QI=26.8 \pm 1.1$ and $\widehat{QI}=24.3\pm 0.8$. These results clearly outperform those provided by previously analyzed clustering methods. Next section assesses these results across different ICA decompositions and subjects.

## Results

For the assessment of the results, this section computes the outputs of the clustering algorithms across 10 ICA decompositions for the same group of subjects and, after that, for 10 different groups of subjects.

**Fig. 8:**
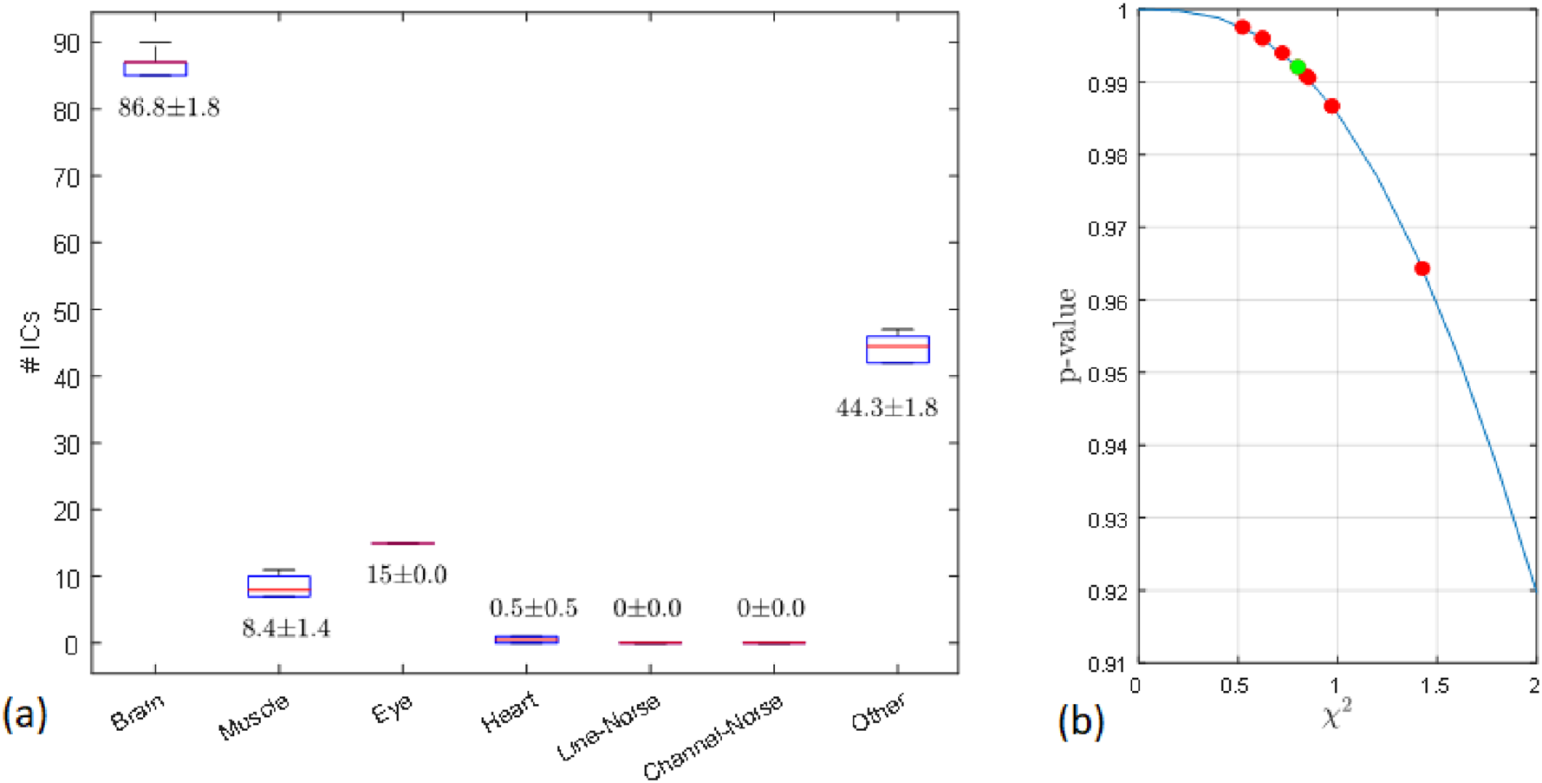
AMICA shows great stability when labels assigned by ICLabel are analyzed. **a** Boxplot of the labels. **b** $\chi ^2$ scores and the corresponding *p*-values for the distribution of the assigned labels (the green dot indicates the decomposition used in the previous section)

Before assessing the results of the clustering algorithm, the reliability of the AMICA decompositions must be analyzed. This reliability is evaluated here by analyzing the results of ICLabel across the 10 ICA decompositions. Figure 8(a) graphs the boxplot of the assigned label. The results show great stability (narrow boxes) which is confirmed when performing a $\chi ^2$ square test for the observed distributions of the given labels, taking the means as the expected values and six degrees (seven possible labels minus 1) of freedom (see Fig. 8(b); the *p*-values are above 95% (the green dot indicates the decomposition used in the previous section). Building upon this stability, a new CVI (*RIm*), inspired by *RI* and with values between 0 and 1, is included regarding the ICs labelled as eyes by ICLabel, and computed as follows:

12 $$\begin{aligned} RIm={\vert C_{eye} \vert } \cdot \sum \limits _{C_i \in S_{eye}}{\frac{1}{\vert C_i \vert }} \end{aligned}$$

where $C_{eye}$ denotes the eye-cluster generated by ICLABEL, and $S_{eye}$ the set of clusters $C_i$ in the evaluated clustering such that $C_i \cap C_{eye} \ne \emptyset$.

Then, Tables 1 and 2 collect the results across ICA decompositions and groups of subjects, respectively. The relative positions between the clustering methods remain: the proposed method obtains the best results, followed by CORRMAP and K-means and K-meansC, with similary performances. As expected, the variations of the results are larger for the subject assessment than for the ICA assessment. However, we note that performances of CORRMAP improves when different groups of subjects are tested. This can be explained because the number of clusters is not an input parameter of this algorithm, which allows that it can be freely adjusted for each group of subjects. Even so, the proposed method still outperforms it clearly for all the CVIs.

**Table 1:** Assessment across ICA decompositions

Algorithm	*QI*	$\widehat{QI}$	$Sh^{1}$	$DB^{1}$	$RIm^{1}$	#clusters	#others
CORRMAP	15.5±1.5	10.9±1.1	15±3	38±2	13±1	13.4±2.6	6.4±1.1
K-means	13.6±1.1	9.3±1	− 12±2	54±2	24±4	12.4±0.5	7.8±5.4
N.Network	10.2±0.8	8.4±1.1	− 14±5	57±3	16±2	10	0
K-meansC	13.6±0.4	8.3±0.5	− 10±2	53±2	22±3	14	0
Proposed Met	**27.6±0.9**	**24±0.7**	**18±2**	** 34±2**	**46±8**	14±0.9	4.9±2.2

$$^{1}$$ $$(\cdot 10^2)$$

**Table 2:** Assessment across Subjects’ groups

	*QI*	$\widehat{QI}$	$Sh^{1}$	$DB^{1}$	$RIm^{1}$	#clusters	#others
CORRMAP	20.9±3.9	14.7±3.3	− 10±5	42±3	15±5	15.8±2.3	7.3±2.1
K-means	13.7±2	9.4±2	− 11±4	53±3	16±5	12.9±0.3	1.6±2.1
N.Network	8.7±2.5	7±3	− 15±6	61±3	12±6	9.7±0.6	1.4±3.8
K-meansC	12.8±2.2	7.6±2.3	− 13±4	54±3	17±5	14	0
Proposed Met	**28±2.3**	**25±2.2**	**17±3**	** 36±3**	**28±8**	13.9 ±1.2	3.3 ±1.1

$$^{1}$$ $$(\cdot 10^2)$$

## Discussion

Clustering of scalp maps is proved to be an effective way to identify relevant source components for ASSR EEGs. This paper proposes a hybrid CGA that dramatically outperforms clustering algorithms provided by EEGLAB; namely, K-means, Neural Network, K-meansCluster and CORRMAP. It consists of a pre-clustering phase based on spectral clustering, which allows a direct adaptation from the pairwise absolute correlation coefficients to similarity graphs, followed by a genetic optimization phase. This optimization phase minimizes a cost function to determine the final partitions, including the number of clusters and the elements which are not assigned to any cluster. This phase implies the computation of centroids that is based on another GA that estimates the polarities of the components. The performances of the proposed algorithm have been evaluated using specific metrics and assessed across different ICA decompositions and groups of subjects, resulting in the proposed algorithm reaching the best results.

## Conclusions

We described in this paper the complete clustering process of topographic maps of the scalp obtained with EEG. The most challenging aspect of this clustering is that traditional euclidean-distance based methods and metrics cannot be directly applied here so they have to be adapted to the use of the absolute correlation coefficient as the similarity measure. Thus, spectral-clustering is combined with genetic optimization to propose a novel hybrid clustering algorithm. We evaluated the use of this algorithm using specifically adapted metrics and concluded that it outperforms significantly the baseline clustering methods. A better clustering of scalp maps should result in a simpler identification of brain-generated processes, so we hope that this work can help researchers working in the field of ASSR EEGs to associate obtained topographic scalp maps with the corresponding populations of interest.

## Declarations

### Data Availability

Datasets generated during and/or analyzed during the current study are not publicly available due to individual privacy but they could shared, totally or partially, upon reasonable request.

### Code Availability

Code used during the current study is not publicly available but it could shared, totally or partially, upon reasonable request.
